# Supplementary material for: Polyketide synthase gene clusters in the ericoid mycorrhizal fungus Oidiodendron maius: genome-wide prediction, expression profiling, and functional characterization of knockout mutants
Source: Front Plant Sci. 2026 Sep 16;17:1924430. doi: 10.3389/fpls.2026.1924430 (PMC13623903; doi:10.3389/fpls.2026.1924430)
Supplement: Supplementary file 1 [file SupplementaryFile1.zip › Supplementary Materials/Supplementary Folder 1.zip!/Oidma1_AssemblyScaffolds_Repeatmasked/knownclusterblast/cluster27/jgi.p_Oidma1_43476_mibig_hits.html]

| MIBiG Protein | Description | MIBiG Cluster | MiBiG Product | % ID | % Coverage | BLAST Score | E-value |
| --- | --- | --- | --- | --- | --- | --- | --- |
| AGA37278.1 | FAD\_monooxygenase | BGC0000819 | Paraherquamide\_biosynthetic\_gene\_cluster | 31 | 94.8051948052 | 217 | 2.7e-56 |
| CBF90103.1 | conserved\_hypothetical\_protein | BGC0001271 | Emericellin\_biosynthetic\_gene\_cluster | 31 | 101.515151515 | 217 | 4.6e-56 |
| CCE67072.1 | monooxygenase | BGC0001242 | Fusarubin\_biosynthetic\_gene\_cluster | 29 | 98.7012987013 | 215 | 1.3e-55 |
| EAL89047.1 | MAK1-like\_monooxygenase,\_putative | BGC0000355 | Fumiquinazolines\_biosynthetic\_gene\_cluster | 31 | 87.6623376623 | 212 | 1.1e-54 |
| CAP96443.1 | unnamed | BGC0000420 | Roquefortine\_C\_/\_meleagrin\_biosynthetic\_gene\_cluster | 30 | 95.670995671 | 201 | 2e-51 |
| ADM34142.1 | FAD\_binding\_domain\_protein | BGC0001084 | Notoamide\_/\_stephacidin\_biosynthetic\_gene\_cluster | 29 | 93.0735930736 | 186 | 6.6e-47 |
| AGC83580.1 | FAD\_monooxygenase | BGC0000818 | Notoamide\_biosynthetic\_gene\_cluster | 30 | 93.29004329 | 186 | 8.7e-47 |
| AGC83573.1 | FAD\_monooxygenase | BGC0000818 | Notoamide\_biosynthetic\_gene\_cluster | 28 | 95.2380952381 | 183 | 7.3e-46 |
| ADM34135.1 | FAD\_binding\_domain\_protein | BGC0001084 | Notoamide\_/\_stephacidin\_biosynthetic\_gene\_cluster | 28 | 86.3636363636 | 166 | 7.1e-41 |
| ADY16690.1 | TqaE | BGC0001142 | Tryptoquialanine\_biosynthetic\_gene\_cluster | 27 | 99.5670995671 | 159 | 1.1e-38 |
| CDF96615.1 | COG0654:\_2-polyprenyl-6-methoxyphenol\_hydroxylase\_and\_related\_FAD-dependent\_oxidoreductases | BGC0001149 | Brabantamide\_biosynthetic\_gene\_cluster | 29 | 77.7056277056 | 113 | 5.5e-25 |
| EED18000.1 | monooxygenase,\_putative | BGC0000154 | Stipitatic\_acid\_biosynthetic\_gene\_cluster | 22 | 57.3593073593 | 64 | 3.8e-10 |
